# Contemporary Management of Uterine Fibroids

**DOI:** 10.3390/jcm15124632

**Published:** 2026-06-15

**Authors:** Olga Połukord, Wiktoria Jędrzejak, Patrycja Loba, Maria Depczyńska, Zuzanna Radziszewska, Dobrochna Stachecka, Maciej Wilczak, Karolina Chmaj-Wierzchowska

**Affiliations:** 1 Department of Maternal and Child Health and Minimally Invasive Gynecologic Surgery, Poznan University of Medical Sciences, 60-701 Poznan, Poland; mwil@ump.edu.pl; 2 The Student of Faculty of Medicine, Poznan University of Medical Sciences, 60-701 Poznan, Poland; wiktoria.jedrzejak@onet.pl (W.J.); patrycja.loba@outlook.com (P.L.); mariadepczynska14@gmail.com (M.D.); z.radziszewska18@gmail.com (Z.R.); dobrochnas@gazeta.pl (D.S.); 3 Laboratory of Advanced Interventional Therapies in Gynecology and Urogynecology, Poznan University of Medical Sciences, 60-701 Poznan, Poland

**Keywords:** uterine fibroids, leiomyoma, uterine artery embolization, radiofrequency ablation, high-intensity focused ultrasound, myomectomy, hysterectomy, hormonal therapy, minimally invasive treatment, quality of life

## Abstract

**Background**: Uterine fibroids (leiomyomas) are the most common benign tumors in women of reproductive age and represent a significant cause of abnormal uterine bleeding, pelvic pain, infertility, and reduced quality of life. Contemporary management has evolved toward individualized, uterus-sparing approaches, incorporating pharmacological and minimally invasive strategies alongside traditional surgical methods. **Methods**: This narrative review was conducted based on a comprehensive search of PubMed/MEDLINE, Scopus, and Web of Science databases for studies published between January 2010 and December 2025. The search strategy combined Medical Subject Headings (MeSH) and free-text terms related to uterine fibroids and their management. Eligible studies included clinical trials, systematic reviews, and meta-analyses focusing on pharmacological, minimally invasive, and surgical treatments in adult women. The review was prepared in accordance with the Scale for the Assessment of Narrative Review Articles (SANRA) recommendations to improve methodological transparency and quality of reporting. **Results**: A total of 97 studies were included in the qualitative synthesis. Minimally invasive techniques, including uterine artery embolization (UAE), radiofrequency ablation (RFA), and high-intensity focused ultrasound (HIFU), demonstrate high efficacy in symptom control and improvement of quality of life, with shorter recovery times and lower complication rates compared to conventional surgery. However, their impact on fertility remains variable and requires careful patient selection. Pharmacological therapies, particularly GnRH analogues and antagonists, effectively reduce bleeding and fibroid volume, although their long-term use is limited by side effects. **Conclusions**: The management of uterine fibroids should be individualized, taking into account symptom severity, fibroid characteristics, patient age, and reproductive plans. Minimally invasive and pharmacological treatments represent effective alternatives to surgery in appropriately selected patients, while surgical approaches remain essential in advanced or refractory cases. Future research should focus on optimizing personalized treatment strategies and evaluating long-term outcomes, particularly regarding fertility and recurrence.

## 1. Introduction

Uterine fibroids are benign lesions that originate from the smooth muscle tissue of the uterus. Their growth is primarily stimulated by sex hormones [1]. The development of uterine fibroids can be attributed to progesterone-mediated proliferation of mutated myometrial stem cells [2]. The influence of sex hormones makes them the most prevalent form of benign tumors among women of reproductive age; however, they are rarely observed before menarche and tend to regress after menopause [1,3].

Current estimates suggest that approximately 70% of women of reproductive age are affected by uterine fibroids [4]. The prevalence of uterine fibroids is higher among women of African descent [2]. Additional risk factors include nulliparity or a first pregnancy during the third decade of life, early onset of menarche and late menopause, caffeine and alcohol consumption, red meat-rich diet, obesity, and hypertension [3,5]. Genetic factors and vitamin D deficiency also play a role in uterine fibroid development [6]. Uterine fibroids are often asymptomatic and are frequently detected incidentally during transvaginal ultrasonography or magnetic resonance imaging. The symptoms experienced by patients may vary according to the number, size, and location of fibroids [4]. Consequently, the presence of uterine fibroids may cause pressure-related complaints such as a sensation of pelvic pressure, back pain and/or lower abdominal pain, and bloating. The compression of adjacent organs, such as the urinary bladder or intestines, may lead to urinary frequency and constipation. The most frequently reported symptom is abnormal uterine bleeding, often associated with iron deficiency and anemia. Dysmenorrhea and sexual dysfunction may also occur in affected individuals [3,4,5,6]. Patients with severe symptoms experience a marked decline in their quality of life. Uterine fibroids also contribute to infertility and pregnancy-related complications [1,7]. They occur in approximately 10% of pregnant women [8] and are associated with complications such as an increase in the risk of preterm birth, cesarean delivery, placenta previa, miscarriage, placental abruption, and postpartum hemorrhage [9,10]. In recent years, the incidence of these complications has been increasing due to the increased maternal age at the time of childbearing [10]. Minimally invasive techniques, including uterine artery embolization (UAE), radiofrequency ablation (RFA), and high-intensity focused ultrasound (HIFU), demonstrate high efficacy in symptom control and improvement of quality of life, with shorter recovery times and lower complication rates compared to conventional surgery. However, their impact on fertility remains variable and requires careful patient selection. Despite the substantial body of literature on uterine fibroids, most available reviews focus on individual treatment modalities or specific aspects of disease management. Given the rapid development of novel pharmacological agents and minimally invasive uterus-sparing techniques, there is a growing need for an updated and clinically oriented synthesis of current evidence. The aim of this narrative review is to provide a comprehensive overview of contemporary management strategies for uterine fibroids, integrating pharmacological, minimally invasive, and surgical approaches within a unified framework. Particular emphasis is placed on treatment efficacy, safety, fertility preservation, recurrence risk, and quality-of-life outcomes to support individualized patient-centered decision-making in contemporary clinical practice.

## 2. Materials and Methods

This study was conducted as a narrative review of the literature focusing on contemporary approaches to the management of uterine fibroids, including pharmacological, minimally invasive, and surgical treatment strategies. The review was prepared in accordance with the Scale for the Assessment of Narrative Review Articles (SANRA) recommendations to improve methodological transparency and quality of reporting.

A comprehensive search of electronic databases, including PubMed/MEDLINE, Scopus, and Web of Science, was performed to identify relevant articles published between January 2010 and December 2025. The search strategy combined Medical Subject Headings (MeSH) and free-text terms using Boolean operators. The following keywords and their combinations were applied: (“uterine fibroids” OR “uterine leiomyoma” OR “leiomyoma” OR “leiomyomata”) AND (“uterine artery embolization” OR “UAE” OR “radiofrequency ablation” OR “RFA” OR “high-intensity focused ultrasound” OR “HIFU” OR “focused ultrasound surgery” OR “MRgFUS” OR myomectomy OR hysterectomy OR “GnRH agonists” OR “GnRH antagonists” OR relugolix OR linzagolix OR “ulipristal acetate” OR “hormonal therapy” OR “medical therapy” OR “conservative treatment” OR “minimally invasive treatment” OR “fertility preservation” OR “quality of life” OR recurrence OR “symptom control” OR “uterus-sparing treatment”).

Additional manual screening of reference lists from relevant systematic reviews, meta-analyses, and international guideline recommendations was also performed to identify potentially eligible studies.

Studies were eligible for inclusion if they met the following criteria:

1. Original clinical studies, randomized controlled trials, cohort studies, systematic reviews, or meta-analyses;
2. Publications addressing pharmacological, minimally invasive, or surgical management of uterine fibroids;
3. Studies involving adult women;
4. Articles published in peer-reviewed journals and available in English.

Exclusion criteria comprised case reports, conference abstracts, editorials, non-peer-reviewed articles, studies lacking clinical relevance, and publications focusing exclusively on experimental or animal models.

The study selection process was conducted in two stages. First, titles and abstracts were screened independently to identify potentially relevant studies. Subsequently, full-text articles were assessed for eligibility. Any discrepancies during the selection process were resolved through discussion and consensus among the authors. Preference was given to high-quality evidence, including randomized controlled trials, systematic reviews, meta-analyses, large cohort studies, and recommendations from international professional societies. The study selection process was conducted independently by five reviewers (W.J., P.L., M.D., and Z.R., D.S.). Titles and abstracts were screened independently to identify potentially relevant studies. Subsequently, full-text articles were assessed for eligibility by the same reviewers. Any discrepancies during the selection process were resolved through discussion and consensus, with consultation of senior authors (O.P., K.C.-W. and M.W.) when necessary. This information has been added to the Materials and Methods section to improve transparency and reproducibility of the review process.

Data extraction was performed using a standardized data collection form developed by the authors. For each eligible study, the following information was extracted: study design, sample size, patient population, fibroid characteristics, treatment modality, primary clinical outcomes, treatment efficacy, safety outcomes, fertility-related outcomes, recurrence rates, and quality-of-life measures when available. Extracted data were reviewed and compared across studies to identify areas of consistency and divergence. The qualitative synthesis was conducted using a thematic narrative approach. Studies were grouped according to treatment modality (pharmacological, minimally invasive, and surgical treatments). Within each category, findings were further organized according to key clinical domains, including symptom control, fibroid volume reduction, safety profile, fertility outcomes, recurrence risk, and quality-of-life impact. The evidence was then compared and interpreted descriptively, with particular emphasis on identifying common findings, differences between treatment strategies, and clinically relevant trends across the literature.

Due to the narrative nature of the review and the heterogeneity of included studies, no formal meta-analysis or validated risk-of-bias assessment was performed. Data were synthesized qualitatively and grouped according to treatment modality, with particular emphasis on treatment efficacy, safety profile, fertility outcomes, recurrence risk, and quality-of-life implications.

## 3. Results

A total of approximately 875 records were initially identified through database searching and manual screening of reference lists. Following the application of the predefined inclusion and exclusion criteria, 97 publications were included in the final qualitative synthesis (Supplementary Table S1).

The included studies were published between 2001 and 2026, with the majority appearing within the last decade, reflecting the rapidly evolving landscape of uterine fibroid management. The evidence base comprised systematic reviews and meta-analyses, randomized and prospective clinical studies, observational cohort studies, retrospective analyses, narrative reviews, and international guideline recommendations. Geographically, the studies originated predominantly from Europe, North America, and Asia, with several multinational collaborative publications also included.

As summarized in Supplementary Table S1, the identified literature covered a broad spectrum of uterine fibroid management strategies. Thematically, studies were categorized into pharmacological therapies, minimally invasive procedures (including uterine artery embolization, radiofrequency ablation, and high-intensity focused ultrasound), surgical management (myomectomy and hysterectomy), fertility and pregnancy outcomes, and patient-reported quality-of-life outcomes.

Overall, the qualitative synthesis demonstrated that minimally invasive treatments are associated with significant symptom relief and improvement in quality of life in appropriately selected patients, while surgical interventions remain essential for women with severe symptoms, large fibroids, infertility, or treatment failure. Pharmacological therapies provide effective symptom control and may reduce fibroid volume, although long-term efficacy and tolerability vary according to the therapeutic agent. Due to the narrative design of this review and the heterogeneity of the included studies, the findings should be interpreted with consideration of potential methodological differences and publication bias.

## 4. Treatment Methods for Uterine Fibroids

### 4.1. Minimally Invasive Treatment Methods for Uterine Fibroids

Minimally invasive interventional radiology techniques provide effective treatment options for patients who prefer to avoid surgical management. These techniques focus on uterus-sparing approaches, primarily for preserving fertility or for application in cases where surgical treatment may be contraindicated. Compared to conventional surgical methods, these techniques are associated with a lower risk of complications and a shorter post-procedural recovery period [11].

Clinical methods for treating uterine fibroids include uterine artery embolization (UAE, often referred to as uterine fibroid embolization [UFE]), uterine artery occlusion (UAO), radiofrequency ablation (RFA), high-intensity focused ultrasound (HIFU), and magnetic resonance-guided focused ultrasound (MRgFUS) [6].

#### 4.1.1. Uterine Artery Embolization (UAE)

Uterine artery embolization (UAE) is a highly effective intervention, with therapeutic efficacy comparable to that of surgical treatment [6]. It involves the selective occlusion of blood vessels supplying the fibroid by using an embolic material introduced through catheterization of the uterine arteries. The procedure is performed under local anesthesia or conscious sedation [11]. Vascular occlusion leads to a reduction in fibroid volume due to ischemic necrosis, as fibroids, unlike normal myometrium, lack the capacity for collateral circulation [12]. This method shows notable efficacy, as evidenced by a reduction in symptom severity and improvement in quality of life in approximately 80% of patients [13]. Compared to patients who undergo myomectomy, those treated with this method experience fewer postoperative complications, a shorter hospital stay, and a reduced rate of readmissions. However, over the long term, there is a higher risk of requiring hysterectomy and other surgical interventions; therefore, treatment should be considered on an individual basis [14]. Uterine artery occlusion, which involves the blocking of fibroid vascularization by ligating the corresponding vessel through laparoscopic techniques, has largely been replaced by radiological embolization procedures [15].

This method is particularly recommended for patients in whom uterine preservation is important, but not in the context of fertility preservation. It facilitates a reduction in fibroid volume by approximately 50–70% and improvement of bleeding symptoms in 80–90% of patients [16]. The main indications include symptomatic, multiple fibroids at various locations (intramural and subserosal), particularly large lesions (>7–8 cm) [17,18]. UAE is suboptimal or contraindicated in patients actively planning pregnancy and in patients with submucosal fibroids FIGO types 0–1, suspected malignancy, and active pelvic infection [18,19,20]. Risks associated with this procedure include the so-called post-embolization syndrome (pain, cramping, nausea, discomfort, and fever lasting several days) [21]. Amenorrhea or premature menopause may occur. The impact of UAE on future pregnancy is uncertain; therefore, its potential effect on fertility should be considered [22].

#### 4.1.2. Radiofrequency Ablation (RFA)

Another minimally invasive technique for treating uterine fibroids is radiofrequency ablation (RFA). This method involves the application of energy, as alternating current, to a targeted area identified using ultrasound through an electrode, which converts it into thermal energy. The resulting high temperature of more than 100 °C induces coagulative necrosis in the tissues [23,24]. Depending on the type and anatomical location of the fibroid, three main access routes are available: transcervical, transvaginal, or laparoscopic. Transvaginal radiofrequency ablation (TV-RFA) has yielded favorable outcomes in terms of effective reduction in fibroid volume and associated symptom severity [25,26]. A two-year follow-up study showed that TV-RFA provides sustained fibroid volume reduction with high safety and a low need for repeat surgical interventions [27]. Retrospective analyses indicate that pregnancies in patients following TV-RFA show outcomes comparable to those in the general population [25,28]. Because RFA is a relatively new technique, further studies are required to validate the current findings and determine its long-term efficacy.

RFA is used for treating a broad spectrum of uterine fibroids (FIGO types 1–5). Large-scale studies such as FAST-EU and SONATA have shown considerable alleviation of bleeding (in 80–90% of patients) and an improvement in quality of life, with a low reintervention rate (8–10% within 3–5 years) and a reduction of approximately 40–60% in fibroid volume [29,30,31,32]. The Sonata TFA system can treat most non-pedunculated fibroids, including submucosal lesions as well as larger or deeper uterine fibroids [31]. RFA is particularly effective for lesions up to approximately 5–7 cm in diameter [30]. The advantages of TFA include the absence of uterine scarring, short procedural time, rapid recovery, and a low rate of postoperative complications [32]. An additional advantage is that it is not contraindicated in women planning pregnancy and does not significantly affect ovarian reserve [33,34].

Transvaginal radiofrequency ablation (TV-RFA) is a particularly noteworthy innovative technique. This method enables the treatment of submucosal fibroids, intramural fibroids with a submucosal component, and some intramural fibroids without contact with the endometrium [29]. TV-RFA allows precise ablation without abdominal or uterine incisions or vascular occlusion, with a short procedure time (approximately up to 30 min) and minimal postoperative trauma [29]. A substantially greater reduction in fibroid size and volume has been observed in patients with Funaki type II fibroids. This method is contraindicated for patients with presence or suspicion of malignancy, acute infection, inflammation at the treatment site, and coagulation disorders [35].

#### 4.1.3. High-Intensity Focused Ultrasound (HIFU/FUS)

High-intensity focused ultrasound (HIFU), also known as focused ultrasound (FUS), is recognized as a minimally invasive method for treating uterine fibroids. This thermal ablation technique involves precise focusing of an ultrasound beam using either ultrasound guidance (USg-HIFU) or magnetic resonance imaging guidance (MRg-HIFU) to heat the target tissue to approximately 65 °C and induce local coagulative necrosis [11]. HIFU is a promising method due to a lower risk of complications compared to surgical management, substantial improvement in quality of life, and decline in symptom severity [36]. Studies indicate that, unlike UAE, HIFU does not adversely affect ovarian reserve [37]. Moreover, this method has shown good outcomes in overweight and obese women [38]. However, further studies are required to assess the incidence of complications such as skin burns, obstetric outcomes, and the need for reintervention [39].

**Figure 1:**
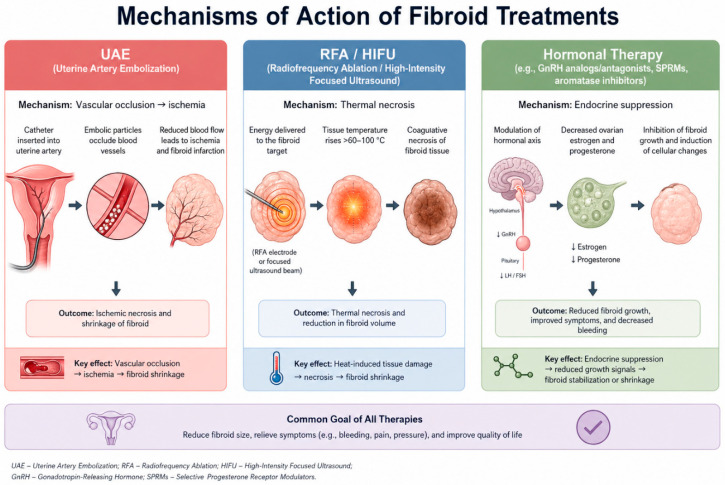
Mechanisms of action of fibroid treatments. The figure was generated with the assistance of ChatGPT (OpenAI, GPT-5.3) based on the authors’ input and specifications.

Optimal results for HIFU are observed in patients presenting with single fibroids who are considered ineligible for surgical treatment [40]. Although UAE and HIFU are recognized as effective methods, they are primarily indicated for treating intramural and subserosal (broad-based) fibroids. However, HIFU has limitations, including a restricted effectiveness in fibroid volume reduction (only approximately 30–50%), which is associated with a higher reintervention rate [41]. During patient selection, factors such as ultrasound accessibility of the fibroid and the presence of scars must be considered [40]. The application of UAE, HIFU, or RFA remains controversial in patients suffering from adenomyosis, endometriosis, and/or ovarian cysts (Figure 1).

Are minimally invasive methods suitable for every patient? Minimally invasive methods for treating uterine fibroids allow selective destruction of fibroids and are increasingly being embraced by both patients and physicians.

This approach not only offers a greater chance of preserving fertility, but it may also serve as an alternative treatment for tumors not eligible for surgical management. In recent years, these methods have undergone dynamic advancements, revealing many new therapeutic possibilities; however, their selection and use require a thorough and individualized assessment of the patient [6]. The characteristics of minimally invasive techniques are summarized in Table 1.

### 4.2. Invasive Methods for the Treatment of Uterine Fibroids

#### 4.2.1. Hysterectomy

Hysterectomy is the most prevalent surgical intervention globally for treating uterine fibroids, enabling complete and permanent resolution of fibroid-related symptoms and prevention of recurrence. Radical surgery is deemed appropriate only for patients who have completed childbearing and have provided consent to uterine removal [42]. Similar to other surgical interventions, hysterectomy is associated with the risk of intra- and postoperative complications, such as urinary tract or bowel injury, hematoma formation, and infections [43]. Other long-term complications include an association with an increased incidence of cardiovascular events, certain cancers, premature menopause, and depression [42]. Despite these risks, hysterectomy remains a commonly used surgical procedure due to the lowest risk of reintervention, whereas the reintervention rate after myomectomy and UAE is 15–20% [44,45].

Abdominal hysterectomy is performed in situations where minimally invasive techniques cannot be applied, such as a significantly enlarged uterus, multiple and large fibroids, and severe symptoms [46,47].

Laparoscopic hysterectomy is currently the most commonly chosen surgical approach in clinical practice. Compared to open hysterectomy, it is associated with reduced intraoperative blood loss, a shorter hospital stay, and a lower mortality rate, while maintaining similar overall complication rates [47,48]. Laparoscopic hysterectomy is recommended when the uterine size does not exceed that of a 13- to 14-week pregnancy.

Vaginal hysterectomy is considered a less invasive procedure than the abdominal approach; however, a key limitation is fibroid size. Preoperative treatment with GnRH analogues may be used to reduce tumor volume [49,50]. This type of hysterectomy may be associated with higher postoperative pain compared to laparoscopic techniques [51]. Robot-assisted laparoscopic hysterectomy is associated with a lower mortality rate, fewer complications, and a shorter postoperative hospital stay compared to other surgical techniques [45,52]. However, its use is limited by the high costs associated with the acquisition of robotic systems [53].

#### 4.2.2. Myomectomy

Myomectomy is a surgical intervention involving the selective removal of uterine fibroids while preserving the uterus [54]. This approach facilitates uterus-sparing treatment, which is particularly important for women of reproductive age who wish to maintain fertility [55]. In cases where fibroids are associated with adverse reproductive outcomes, myomectomy increases the likelihood of postoperative pregnancy in more than 50% of patients [56]. The procedure can be performed using hysteroscopic, laparoscopic, robotic, or conventional open laparotomy approaches [57].

Abdominal myomectomy is an appropriate method for removing multiple, deeply located, and large fibroids. Conventional laparotomy provides direct and comprehensive access to the entire uterus [58]. This approach is considered safer for women planning future pregnancy, as it is associated with a low risk of subsequent uterine rupture, although its disadvantages include a higher risk of adhesions, larger incisions, and more severe postoperative pain [59,60].

Laparoscopic myomectomy shows optimal results for patients with intramural and subserosal fibroids and in women experiencing infertility [61]. In cases where large fibroids necessitate morcellation, a laparoscopic retrieval bag (endobag) should be used to prevent tissue dissemination [62,63]. Donnez et al. proposed the following criteria for patient selection for laparoscopic myomectomy: (1) fibroids are <10–12 cm in size, (2) there are no more than 3–4 intramural fibroids, or (3) there are intramural fibroids of >3–5 cm that distort the uterine cavity in patients treated for infertility. Contraindications include very large or numerous fibroids, suspicion of malignancy, and severe systemic diseases [64].

Robot-assisted myomectomy may be used in technically challenging cases. Compared to conventional laparoscopy, robotic assistance enables more precise movements, facilitating more effective fibroid dissection and tissue suturing [65]. The advantages include a lower complication rate, reduced blood loss, and less postoperative pain [66,67].

Hysteroscopic myomectomy involves fibroid resection using an electrosurgical loop under direct visual control. This technique is primarily used for removing submucosal fibroids and is characterized by a low risk of complications [68,69]. This method shows the most favorable outcomes for patients diagnosed with FIGO type 0–1 fibroids, particularly those experiencing heavy menstrual bleeding, infertility, or recurrent miscarriages [70,71]. The advantages include high efficacy in reducing bleeding in 90–95% of patients with FIGO 0–1 fibroids and no uterine scars [70,71]. For patients requiring extensive resection, there is a risk of intrauterine adhesions [72]. Contraindications include active infection or suspected malignancy. A comparison of surgical treatment options is presented in Table 2.

### 4.3. Hormonal Treatment of Uterine Fibroids

Hormonal treatment of uterine fibroids enables symptom control and tumor mass reduction. It is primarily indicated for women who wish to preserve their fertility. The therapy is based on the modulation of estrogen and progesterone activity, necessitating a tailored selection process and a specified duration of treatment [6]. Preoperative hormonal therapy can be used to reduce perioperative complications [73].

#### 4.3.1. Progestogens/Levonorgestrel-Releasing Intrauterine System (LNG-IUS)

Progestogens, both natural and synthetic, may influence the growth of uterine fibroids through two mechanisms. Progesterone stimulates epidermal growth factor (EGF), which promotes fibroid development, while inhibiting IGF-1, which may limit fibroid growth [74]. Previous studies have shown that the use of LNG-IUS may reduce blood loss in women with fibroids; however, there is a lack of substantial evidence that progestogens significantly reduce fibroid volume [75]. Systematic reviews indicate that the available studies are of low methodological quality, and there is evidence that progesterone and progestogens may influence fibroid development, raising concerns regarding their routine use [76].

#### 4.3.2. Gonadotropin-Releasing Hormone (GnRH) Agonists and Antagonists

GnRH agonists and antagonists are an important group of drugs used for treating symptomatic uterine fibroids. Their mechanism of action involves suppression of the hypothalamic–pituitary–gonadal axis, leading to decreased levels of estrogen and progesterone. GnRH agonists initially cause a transient stimulation of receptors, followed by marked hormonal suppression, leading to a reduction in fibroid volume and decreased menstrual bleeding. However, their use is limited by adverse effects related to hypoestrogenism, such as vasomotor symptoms and loss of bone mineral density [77].

GnRH antagonists exert their effects through a direct and reversible blockade of GnRH receptors, enabling rapid suppression of gonadotropin secretion without a “flare” effect. Systematic reviews and meta-analyses have confirmed that GnRH antagonists can reduce both menstrual bleeding and uterine fibroid volume [78]. Linzagolix, an oral medication, is indicated for treating moderate to severe symptoms of uterine fibroids and endometriosis in adult women. The use of add-back therapy improves tolerability and safety of long-term treatment in premenopausal women [78].

#### 4.3.3. Selective Progesterone Receptor Modulators (SPRMs)

Selective progesterone receptor modulators (SPRMs) are a class of compounds that bind to the progesterone receptor and exhibit agonistic, antagonistic, partial, or mixed effects in progesterone-sensitive tissues [73]. Short-term use of SPRMs improves quality of life, reduces menstrual bleeding, and increases the rate of amenorrhea compared to placebo [79].

Ulipristal acetate (UPA) is a selective progesterone receptor modulator used for treating uterine fibroids; its therapeutic actions involve reduction in fibroid volume, improvement of hemoglobin levels, and regulation of menstrual bleeding [80]. It does not cause hypoestrogenic effects; however, its use is associated with a rare but serious risk of liver injury associated with ulipristal acetate, including rare cases progressing to acute liver failure requiring liver transplantation [7]. Furthermore, the updated text emphasizes the importance of careful patient selection and mandatory liver function monitoring in accordance with current regulatory guidance, as well as the need for individualized risk–benefit assessment prior to initiating therapy [81].

#### 4.3.4. Selective Estrogen Receptor Modulators (SERMs)

A systematic review of clinical trials revealed a lack of consistent evidence supporting the efficacy of raloxifene in improving the clinical conditions of patients. The low quality of the available data and heterogeneous results raises questions regarding the role of SERM drugs in treating this pathology, warranting further research [82]. Limited data on the safety profile necessitate caution, particularly due to the potential risk of thromboembolic complications when the drug is used at higher doses [6].

#### 4.3.5. Aromatase Inhibitors

Aromatase inhibitors block the cytochrome P450 enzyme, thereby inhibiting the conversion of androgens to estrogens [4]. They may lead to a reduction in fibroid volume accompanied by a lower frequency of hot flushes compared to that following treatment with GnRH agonists. Despite promising results, current evidence is insufficient to clearly confirm their clinical efficacy and long-term safety. Because of gaps in data and the risk of decreased bone mineral density, these agents are not currently recommended for routine treatment [83].

#### 4.3.6. Relugolix/Estradiol/Norethisterone Acetate

A GnRH receptor antagonist (relugolix) is combined with add-back hormone replacement therapy in a single tablet. It significantly alleviates dysmenorrhea and non-menstrual pelvic pain, with benefits maintained over long-term use, and has a minimal impact on bone loss [84]. It is indicated for treating moderate to severe symptoms of uterine fibroids in adult women of reproductive age [85]. Contraindications include a high risk of thrombotic or thromboembolic disorders, pregnancy, osteoporosis, hepatic impairment or liver disease, hormone-sensitive malignancies, and unexplained abnormal uterine bleeding [85]. Concomitant use of hormonal contraceptives with relugolix/estradiol/norethisterone acetate is not recommended. Pharmacological therapies are outlined in Table 3.

## 5. Quality of Life Implications

### 5.1. Quality of Life in Women with Uterine Fibroids

Uterine fibroids occur in approximately 70% of women, of whom approximately 30% are symptomatic [3]. Hysteroscopic myomectomy has been associated with significant improvement in symptom control and health-related quality of life, particularly through the reduction of abnormal uterine bleeding and the preservation of reproductive potential [86,87]. Both laparoscopic myomectomy and magnetic resonance-guided focused ultrasound (MRgFUS) have been associated with improvements in patients’ quality of life through effective symptom relief [41,88,89]. Reported symptoms include abnormal uterine bleeding, anemia, increased urinary frequency, lower abdominal pain, infertility, and recurrent miscarriages [90,91]. Abnormal uterine bleeding is the most common symptom [90]. Moreover, 30% of patients with fibroids report a reduction in their quality of life [4]. Global and fibroid-specific assessment scales indicate that women with uterine fibroids also experience a psychological burden affecting quality of life, including fear, anxiety, uncertainty, helplessness, depression, and reduced self-esteem.

Patients also reported health-related disability in the domains of physical role functioning, bodily pain, and sexual satisfaction. Patients with fibroids showed lower social functioning scores than women suffering from cardiovascular diseases, diabetes, or breast cancer [92]. Studies have demonstrated that domains related to perceived loss of control over one’s health and future-related concerns were associated with the lowest baseline scores, reflecting the poorest symptom-related quality of life.

Uterine fibroids are the most common indication for hysterectomy [92]. Women undergoing abdominal hysterectomy report the lowest quality of life and the most severe symptoms compared to those treated with uterus-preserving methods. The greatest improvement in quality of life has been observed after laparoscopic hysterectomy [93].

### 5.2. Psychophysical Predispositions Influencing Treatment Decisions

Hysterectomy remains one of the most commonly performed definitive treatment options for symptomatic uterine fibroids, particularly among women who do not desire future fertility [94]. Regardless of age and race, the predominant determinants guiding women’s decision regarding surgical treatment are symptom relief and concern about a potential malignant process. The additional factors considered are the need for re-treatment and treatment-related complications. Stratification according to age revealed that women under 40 years of age placed greater emphasis on the possibility of future childbearing after treatment and therefore more often opted for uterus-preserving treatment methods [95].

Robot-assisted laparoscopic hysterectomy may be associated with fewer perioperative complications and shorter hospital stay in selected patient populations; however, current evidence does not clearly demonstrate superiority over conventional laparoscopic approaches, and its use remains limited by the high costs associated with robotic systems. They also expect more reliable information about uterus-preserving treatment methods. The absence of shared decision-making reduced trust in the physician, whereas greater collaboration led to higher acceptance of the chosen therapy [96].

The management of uterine fibroids has undergone a significant transformation in recent decades, shifting from a predominantly surgical paradigm toward a more individualized, multimodal approach. The findings of this review indicate that minimally invasive techniques—including UAE, RFA, and HIFU—represent effective alternatives to surgery for appropriately selected patients, particularly those wishing to preserve the uterus or avoid the risks of conventional surgery.

Uterine artery embolization remains one of the most widely used minimally invasive procedures, with high rates of symptom relief and patient satisfaction. However, concerns regarding its impact on fertility and the risk of premature menopause necessitate careful patient counseling, particularly in women of reproductive age [6,11]. Radiofrequency ablation, especially the transcervical approach using the Sonata system, has shown promising results in terms of fibroid volume reduction and quality of life improvement, with a favorable safety profile and low reintervention rates [29,30,32]. The absence of uterine scarring is a particular advantage for women planning future pregnancies.

High-intensity focused ultrasound, while entirely non-invasive and associated with minimal complications, is constrained by lower volume reduction rates and a higher risk of reintervention compared to other modalities [41]. Patient selection—based on fibroid size, location, and accessibility—remains critical for optimizing outcomes. The role of MRI guidance in improving precision has been demonstrated, but limited availability and cost restrict its widespread application.

Surgical approaches, particularly myomectomy, continue to play a central role in the management of fibroids associated with infertility or recurrent pregnancy loss. Laparoscopic and hysteroscopic myomectomy offer acceptable outcomes with reduced morbidity compared to open surgery, provided that appropriate patient selection criteria are applied [57,69]. Hysterectomy remains the definitive treatment and is associated with the lowest risk of symptom recurrence; however, its irreversible nature limits its applicability to women who have completed childbearing [42,44].

Pharmacological therapies, especially GnRH agonists and the newer oral GnRH antagonists such as relugolix, provide effective short-term symptom control and are useful as preoperative adjuncts or stand-alone treatments in women approaching menopause [77,78,84]. The combination of GnRH antagonists with add-back hormone therapy has improved tolerability and expanded the potential duration of treatment. However, long-term pharmacological management remains limited by side effects, incomplete fibroid regression, and symptom recurrence upon discontinuation.

The impact of uterine fibroids on quality of life is substantial and multidimensional, encompassing physical, psychological, and social domains [90,91]. Shared decision-making between patient and clinician—incorporating individual symptom burden, reproductive goals, and treatment preferences—is fundamental to achieving satisfactory outcomes [96]. Evidence suggests that patients who are actively involved in treatment decisions demonstrate greater acceptance of and adherence to the chosen therapy.

A more critical comparison of minimally invasive and surgical treatments highlights significant differences in long-term outcomes, recurrence rates, and cost-effectiveness. Minimally invasive procedures are associated with significant short-term symptom relief and faster recovery; however, they exhibit variable reintervention rates over time depending on the technique and patient selection. This reflects the potential for symptom recurrence or incomplete fibroid eradication in some patients. Surgical treatment, particularly hysterectomy, provides definitive treatment. Myomectomy, while uterine-sparing, is also associated with recurrence rates depending on the duration of follow-up and fibroid characteristics. From a cost-effectiveness perspective, minimally invasive treatments may offer short-term economic benefits due to shorter hospital stays and faster return to daily activities; however, repeated interventions may increase cumulative costs in the long term, potentially offsetting the initial savings. Therefore, the choice of treatment method should consider the balance between immediate perioperative benefits and long-term durability and patient priorities, particularly reproductive goals and willingness to accept the risk of reintervention [97].

## 6. Future Perspectives

Future research should focus on personalized treatment strategies integrating clinical, imaging, and molecular data to improve patient selection and outcome prediction. The development of novel oral therapies with improved safety profiles and sustained efficacy may further reduce dependence on surgical interventions. Long-term prospective studies are needed to establish the fertility and obstetric safety of minimally invasive techniques, particularly RFA and HIFU, in women of reproductive age. Advances in artificial intelligence and high-resolution imaging may also contribute to more precise fibroid characterization and treatment planning. Additionally, biomarker-based approaches to predict fibroid growth, recurrence, and treatment response represent a promising avenue for individualized management.

## 7. Summary

The selection of the appropriate treatment method for uterine fibroids should be individualized and consider the patient’s age, severity of symptoms, size and location of the lesions, and reproductive plans. Minimally invasive methods represent a valuable alternative to surgical treatment, particularly in patients wishing to preserve the uterus. However, they are not suitable for all cases, particularly for patients presenting with large, multiple, or atypically located fibroids. Hysterectomy remains the most effective method for eliminating symptoms, although its radical nature limits its use in women planning future pregnancy. Summary of International Guideline Recommendations for the Management of Uterine Fibroids present Table 4.

Uterine fibroids significantly affect the patient’s quality of life, both physically and psychologically. The most important symptoms include abnormal uterine bleeding, pain, and fertility-related problems. Therapeutic decision-making should be shared with the patient, considering her preferences and providing reliable information on the available treatment options.

This review provides a comprehensive and up-to-date synthesis of pharmacological, minimally invasive, and surgical strategies for uterine fibroid management within a unified clinical framework. It uniquely integrates emerging uterus-sparing technologies and novel medical therapies with direct comparative evaluation of recurrence, fertility outcomes, and long-term effectiveness. By bridging fragmented evidence across disciplines, it offers a decision-oriented approach that better reflects current and evolving clinical practice.

## 8. Limitations

This review is limited by the heterogeneity of the included studies and the lack of long-term data for some minimally invasive techniques. Variability in study design, patient selection criteria, outcome measures, and follow-up duration may have influenced the reported results. In addition, the restriction to English-language publications may have resulted in the exclusion of relevant international evidence, introducing potential language bias. As a narrative review, this study did not employ a formal systematic review methodology or quantitative meta-analysis, which may increase the risk of publication bias and limit the ability to directly compare treatment outcomes across studies. Finally, no formal risk-of-bias assessment was performed, and the findings should therefore be interpreted with appropriate caution.

## 9. Conclusions

1. Uterine fibroids are a significant clinical issue that adversely affects the quality of life of women of reproductive age.
2. Treatment selection should be individualized and based on symptoms, lesion characteristics, and the patient’s reproductive plans.
3. Minimally invasive methods constitute an effective and safe alternative to surgical treatment in appropriately selected patients.
4. Hysterectomy is the most effective method for symptom elimination; however, its use is limited by the loss of fertility.
5. Hormonal treatment is an important component of symptomatic therapy, although it shows limited effectiveness in reducing fibroid volume.
6. Further studies are required to evaluate the long-term efficacy and safety of emerging treatment methods.

## Figures and Tables

**Table 1:** Comparison of Minimally Invasive Techniques for Uterine Fibroids.

Method	Mechanism of Action	Main Indications	Advantages	Limitations	Fertility Considerations
UAE	Occlusion of uterine arteries resulting in ischemic necrosis of fibroid tissue	Multiple or large fibroids (>7–8 cm) in symptomatic patients who do not plan future pregnancy	High efficacy (approximately 80–90% symptom relief); short hospital stay	Post-embolization syndrome; risk of reintervention; potential ovarian dysfunction	Uncertain; generally not recommended for women planning pregnancy
RFA	Thermal coagulative necrosis induced by temperatures exceeding 100 °C	FIGO types 1–5; small-to-medium fibroids (≤5–7 cm)	Minimally invasive; no uterine scar formation; rapid recovery	Limited long-term outcome data; not suitable for all fibroid types	Favorable; reported pregnancy outcomes are comparable to those in the general population
HIFU (US-guided)	Focused ultrasound energy causing thermal ablation (approximately 65 °C)	Solitary and accessible fibroids; patients unsuitable for surgery	Non-invasive; no incisions; low complication rate	Lower fibroid volume reduction (30–50%); higher reintervention rate	Minimal impact on ovarian reserve
MRgFUS	MRI-guided focused ultrasound enabling precise thermal ablation	Selected patients with well-visualized fibroids	High treatment precision; real-time monitoring	Limited availability; high cost; strict patient selection criteria	Limited evidence regarding reproductive outcomes

**Table 2:** Comparison of Surgical Treatment Options.

Method	Main Indications	Advantages	Limitations	Fertility Impact
Abdominal hysterectomy	Severe symptoms, enlarged uterus, and no desire for future fertility	Definitive treatment with no risk of recurrence	Irreversible procedure; surgical risks; potential long-term complications	Eliminates fertility
Laparoscopic hysterectomy	Uterine size ≤ 13–14 gestational weeks	Reduced blood loss; shorter hospital stay; faster recovery	Requires advanced surgical expertise	Eliminates fertility
Vaginal hysterectomy	Smaller uterus and favorable pelvic anatomy	Least invasive approach; cost-effective; rapid recovery	Limited by uterine and fibroid size	Eliminates fertility
Robot-assisted hysterectomy	Complex surgical cases	Enhanced precision and visualization; potentially fewer complications	High cost; limited availability	Eliminates fertility
Myomectomy	Women wishing to preserve fertility or the uterus	Uterine preservation; improved reproductive outcomes	Risk of recurrence and postoperative adhesions	Preserved
Laparoscopic myomectomy	Fibroids ≤ 10–12 cm and limited in number (≤3–4)	Faster recovery; less postoperative pain; shorter hospitalization	Technically demanding; potential risk of uterine rupture during subsequent pregnancy	Preserved
Hysteroscopic myomectomy	Submucosal fibroids (FIGO types 0–1)	High efficacy in controlling abnormal uterine bleeding (90–95%); no abdominal scars	Applicable only to submucosal fibroids	Preserved
Open (abdominal) myomectomy	Large, multiple, or deeply located fibroids	Complete surgical access; suitable for complex cases	Longer recovery period; increased risk of postoperative adhesions	Preserved

**Table 3:** Pharmacological Treatment of Uterine Fibroids.

Drug Class	Mechanism	Effect on Bleeding	Effect on Fibroid Size	Key Limitations
Progestogens/LNG-IUS	Endometrial suppression	↓ bleeding	Minimal	Weak evidence, possible fibroid stimulation
GnRH Agonists	Hypoestrogenism (after initial flare)	↓↓ bleeding	↓↓ size	Bone loss, menopausal symptoms
GnRH Antagonists (e.g., linzagolix)	Direct GnRH receptor blockade	↓↓ bleeding	↓ size	Cost, need for add-back therapy
SPRMs (e.g., ulipristal acetate)	Progesterone receptor modulation	↓ bleeding, amenorrhea	↓ size	Rare hepatotoxicity (risk of liver injury)
SERMs (e.g., raloxifene)	Estrogen receptor modulation	Variable	Unclear	Limited evidence, thromboembolism risk
Aromatase inhibitors	Block estrogen synthesis (inhibit CYP450)	↓ bleeding	↓ size	Bone loss, insufficient long-term data
Relugolix/estradiol/NETA	GnRH blockade + add-back therapy	↓↓ bleeding	↓ size	Contraindicated in thromboembolism, liver disease

**Table 4:** Summary of International Guideline Recommendations for the Management of Uterine Fibroids [98,99,100,101,102,103,104].

Organization	Main Recommendations	Fertility Preservation	Minimally Invasive Techniques	Pharmacological Therapy	Surgical Treatment
FIGO (International Federation of Gynecology and Obstetrics)	Recommends individualized management based on symptom severity, fibroid size/location, age, and reproductive plans. Supports classification of fibroids using the FIGO system.	Myomectomy is preferred in women desiring future pregnancy. Uterus-sparing approaches should be prioritized when feasible.	UAE, RFA, and HIFU may be considered in selected patients; careful counseling regarding reproductive outcomes is required.	GnRH agonists/antagonists and SPRMs may be used for symptom control and preoperative optimization.	Hysterectomy remains definitive treatment for women without reproductive plans.
ACOG (American College of Obstetricians and Gynecologists)	Emphasizes patient-centered and shared decision-making. Treatment depends on symptoms, fertility goals, and patient preference.	Myomectomy is recommended for symptomatic women seeking fertility preservation. UAE should be used cautiously in women desiring pregnancy.	Minimally invasive approaches are preferred whenever appropriate due to lower morbidity and shorter recovery.	Supports use of LNG-IUS, GnRH agonists, oral GnRH antagonists, and hormonal therapies for bleeding control.	Minimally invasive hysterectomy is preferred over abdominal hysterectomy whenever feasible.
ESGE (European Society for Gynaecological Endoscopy)	Encourages minimally invasive and uterus-preserving approaches whenever clinically appropriate.	Hysteroscopic and laparoscopic myomectomy are recommended for fertility preservation depending on FIGO fibroid type.	Strong support for hysteroscopic, laparoscopic, robotic, and image-guided minimally invasive procedures in selected patients.	Pharmacological treatment may be used preoperatively or for symptom management.	Surgical approach should be individualized according to fibroid burden and surgical expertise.
NICE (National Institute for Health and Care Excellence)	Recommends individualized care based on symptoms, fibroid size, and patient preferences. MRI is advised for large or complex fibroids.	Fertility wishes should guide treatment selection and counseling.	UAE is recommended for selected symptomatic patients; counseling regarding fertility uncertainty is necessary.	LNG-IUS is recommended as first-line therapy for heavy menstrual bleeding when appropriate. GnRH analogues may be used short-term preoperatively.	Hysterectomy should be considered only after discussing less invasive alternatives and fertility implications.

## Data Availability

The data presented in this study are available on request from the corresponding author.

## Supplementary Materials

The following supporting information can be downloaded at: https://www.mdpi.com/article/10.3390/jcm15124632/s1.

## Abbreviations

The following abbreviations are used in this manuscript:

UAE	Uterine Artery Embolization
UFE	Uterine Fibroid Embolization
UAO	Uterine Artery Occlusion
RFA	Radiofrequency Ablation
TV-RFA	Transvaginal Radiofrequency Ablation
TFA	Transcervical Fibroid Ablation
HIFU	High-Intensity Focused Ultrasound
FUS	Focused Ultrasound
USg-HIFU	Ultrasound-Guided HIFU
MRgFUS	Magnetic Resonance-Guided Focused Ultrasound
GnRH	Gonadotropin-Releasing Hormone
SPRMs	Selective Progesterone Receptor Modulators
SERMs	Selective Estrogen Receptor Modulators
UPA	Ulipristal Acetate
LNG-IUS	Levonorgestrel-Releasing Intrauterine System
NETA	Norethisterone Acetate
MeSH	Medical Subject Headings
EGF	Epidermal Growth Factor
IGF-1	Insulin-Like Growth Factor 1
FIGO	International Federation of Gynecology and Obstetrics
ACOG	American College of Obstetricians and Gynecologists
ESGE	European Society for Gynaecological Endoscopy
NICE	National Institute for Health and Care Excellence
