# Supplementary material for: Contemporary Management of Uterine Fibroids
Source: J Clin Med. 2026 Jun 15;15(12):4632. doi: 10.3390/jcm15124632 (PMC13301987; doi:10.3390/jcm15124632)
Supplement: Supplementary file 1 [file jcm-15-04632-s001.zip › jcm-4318001-supplementary.pdf]

Characteristics of Included Studies (References 1–97)

Overall: 97 studies published between 2001 and 2026, representing 11 study design categories and authors from more than 25 countries across 5 continents.

Table S1. Time Span of Publication Dates

Period	Years included	n
2001–2005	2001, 2002 (×2)	3
2006–2010	2006 (×2), 2008 (×2), 2010	5
2011–2015	2011 (×3), 2012 (×6), 2013 (×3), 2014 (×2), 2015	15
2016–2020	2016 (×3), 2017 (×5), 2018, 2019 (×3), 2020 (×4)	16
2021–2022	2021 (×7), 2022 (×8)	15
2023–2024	2023 (×8), 2024 (×11)	19
2025–2026	2025 (×17), 2026 (×7)	24
**TOTAL**		**97**

Table S2. Study Design Types

Study type	n	Reference numbers
Narrative / non-systematic review	33	1, 2, 3, 4, 5, 6, 7, 8, 9, 11, 13, 16, 17, 23, 30, 31, 38, 39, 40, 50, 51, 53, 55, 56, 59, 62, 71, 74, 75, 77, 80, 88, 92
Systematic review / meta-analysis	24	10, 14, 22, 25, 26, 29, 33, 34, 35, 41, 43, 44, 46, 47, 48, 49, 54, 57, 58, 60, 61, 76, 89, 97
Retrospective cohort / database study	10	12, 28, 36, 42, 64, 81, 82, 85, 93, 94
Observational / prospective cohort study	12	15, 18, 19, 20, 21, 24, 27, 65, 66, 68, 69, 72
Clinical guideline / expert consensus	7	52, 63, 73, 78, 79, 84, 87
Randomised controlled trial (RCT)	3	32, 67, 83
Cross-sectional / survey study	3	90, 91, 95
Book chapter / textbook entry	2	70, 86
Cost / health-economic analysis	1	37
Qualitative / mixed-methods study	1	96
Case report	1	45
**TOTAL**	**97**	

Note: Studies comprising multiple design elements (e.g. systematic review with meta-analysis) are classified under their primary design category. The total is 97.

Table S3. Geographical Origin of Included Studies

Country / region	n*	Reference numbers
USA	25	1, 3, 5, 7, 11, 16, 26, 28, 30, 32, 35, 36, 40, 43, 67, 70, 74, 79, 82, 84, 88, 93, 94, 95, 96
Poland	8	23, 45, 56, 59, 64, 72, 77, 80

Country / region	n*	Reference numbers
China	8	10, 14, 17, 22, 24, 48, 60, 89
Germany	7	13, 65, 66, 68, 69, 73, 85
Belgium	5	5, 7, 44, 55, 83
France	5	29, 46, 78, 83, 91
Italy	5	4, 39, 43, 50, 86
Spain	5	18, 19, 20, 21, 57
UK	5	11, 32, 61, 62, 87
Canada	4	8, 47, 52, 58
Brazil	3	31, 51, 81
Japan	2	53, 90
New Zealand / Australia	2	33, 34
Serbia	2	6, 12
Taiwan	2	25, 38
Czech Republic	1	15
Finland	1	37
India	1	75
Iran	1	71
Ireland	1	76
Kazakhstan	1	12
Lithuania	1	9
Netherlands	1	92
Norway	1	42
Pakistan	1	27
Philippines	1	41
Saudi Arabia	1	49
Sweden	1	9
Thailand	1	54
Ukraine	1	83
EU (EMA – regulatory)	1	63

** Studies with multi-country authorship teams are counted once per contributing country; the column sum therefore exceeds 97. References 9 (Lithuania/Sweden/Ireland/Spain/Ghana/Uganda), 63 (EU/EMA), 78 (Malta/Portugal/France/Belgium/Morocco), and 83 (Belgium/Ukraine/France) represent multi-country collaborations and are listed under each contributing country individually.*

References

1. De La Cruz, M.S.; Buchanan, E.M. Uterine Fibroids: Diagnosis and Treatment. *Am. Fam. Physician* **2017**, *95*, 100–107.
2. Bulun, S.E.; Yin, P.; Wei, J.; Zuberi, A.; Iizuka, T.; Suzuki, T.; Saini, P.; Goad, J.; Parker, J.B.; Adli, M.; et al. Uterine Fibroids. *Physiol. Rev.* **2025**, *105*, 1947–1988. https://doi.org/10.1152/physrev.00010.2024.
3. Giuliani, E.; As-Sanie, S.; Marsh, E.E. Epidemiology and management of uterine fibroids. *Int. J. Gynaecol. Obstet.* **2020**, *149*, 3–9. https://doi.org/10.1002/ijgo.13102.

4. Vannuccini, S.; Petraglia, F.; Carmona, F.; Calaf, J.; Chapron, C. The modern management of uterine fibroids-related abnormal uterine bleeding. *Fertil. Steril.* **2024**, *122*, 20–30. https://doi.org/10.1016/j.fertnstert.2024.04.041.
5. Dolmans, M.M.; Petraglia, F.; Catherino, W.H.; Donnez, J. Pathogenesis of uterine fibroids: Current understanding and future directions. *Fertil. Steril.* **2024**, *122*, 6–11. https://doi.org/10.1016/j.fertnstert.2024.02.048.
6. Micić, J.; Macura, M.; Andjić, M.; Ivanović, K.; Dotlić, J.; Micić, D.D.; Arsenijević, V.; Stojnić, J.; Bila, J.; Babić, S.; et al. Currently Available Treatment Modalities for Uterine Fibroids. *Medicina* **2024**, *60*, 868. https://doi.org/10.3390/medicina60060868.
7. Donnez, J.; Taylor, H.S.; Marcellin, L.; Dolmans, M.M. Uterine fibroid-related infertility: Mechanisms and management. *Fertil. Steril.* **2024**, *122*, 31–39. https://doi.org/10.1016/j.fertnstert.2024.02.049.
8. Sobel, M.; Hobson, S.; Chan, C. Uterine fibroids in pregnancy. *Can. Med Assoc. J.* **2022**, *194*, E775. https://doi.org/10.1503/cmaj.211530.
9. Ramasauskaite, D.; Purandare, N.; Diaz, I.; Kvederaite-Budre, G.; Beyuo, T.K.; Beyeza-Kashesya, J.; Jacobsson, B. Fibroids and pregnancy. *Int. J. Gynaecol. Obstet.* **2026**, *172*, 51–58. https://doi.org/10.1002/ijgo.70612.
10. Li, H.; Hu, Z.; Fan, Y.; Hao, Y. The influence of uterine fibroids on adverse outcomes in pregnant women: A meta-analysis. *BMC Pregnancy Childbirth* **2024**, *24*, 345. https://doi.org/10.1186/s12884-024-06545-5.
11. Lewis, J.M.; Algurjia, E.; Lumsden, M.A.; Meinhold-Heerlein, I.; Wasson, M.N.; Spiers, A.; Descamps, P. Image-guided therapies for uterine fibroids. *Int. J. Gynecol. Obstet.* **2025**, *171*, 495. https://doi.org/10.1002/ijgo.70493.
12. Garzon, S.; Ukybassova, T.; Terzic, M.; Dotlic, J.; Imankulova, B.; Terzic, S.; Shauyen, F.; Guo, L.; Sui, L. Evaluation of uterine artery embolization on myoma shrinkage: Results from a large cohort analysis. *Gynecol. Minim. Invasive Ther.* **2019**, *8*, 165–171. https://doi.org/10.4103/GMIT.GMIT_50_19.
13. Kröncke, T. An update on uterine artery embolization for uterine leiomyomata and adenomyosis of the uterus. *Br. J. Radiol.* **2023**, *96*, 20220121. https://doi.org/10.1259/bjr.20220121.
14. Peng, J.; Wang, J.; Shu, Q.; Luo, Y.; Wang, S.; Liu, Z. Systematic review and meta-analysis of current evidence in uterine artery embolization vs myomectomy for symptomatic uterine fibroids. *Sci. Rep.* **2024**, *14*, 19252. https://doi.org/10.1038/s41598-024-69754-0.
15. Mara, M.; Kubinova, K.; Maskova, J.; Horak, P.; Belsan, T.; Kuzel, D. Uterine artery embolization versus laparoscopic uterine artery occlusion: Outcomes of a prospective nonrandomized clinical trial. *Cardiovasc. Interv. Radiol.* **2012**, *35*, 1041–1052. https://doi.org/10.1007/s00270-012-0388-y.
16. Lee, B.B.; Yu, S.P. Radiofrequency ablation of uterine fibroids: A review. *Curr. Obstet. Gynecol. Rep.* **2016**, *5*, 318–324. https://doi.org/10.1007/s13669-016-0183-x.
17. Wu, J.; Zhou, Z.; Huang, Y.; Deng, X.; Zheng, S.; He, S.; Huang, G.; Hu, B.; Shi, M.; Liao, W.; et al. Radiofrequency ablation: Mechanisms and clinical applications. *MedComm* **2024**, *5*, e746. https://doi.org/10.1002/mco2.746.
18. Marín Martínez, M.E.; Cruz-Melguizo, S.; Vaquero Argüello, G.; Engels Calvo, V.; De la Cruz Conty, M.L.; Pérez Medina, T. Transvaginal radiofrequency ablation: Therapeutic option for symptomatic uterine fibroids in women with reproductive desires. *F&S Rep.* **2024**, *5*, 320–327. https://doi.org/10.1016/j.xfre.2024.07.001.
19. Ariza, D.E.; García, I.R.; Cabreja, J.A.Á.; Carmona, E.H. Outcomes of transvaginal radiofrequency ablation for symptomatic leiomyomas. *J. Gynecol. Obstet. Hum. Reprod.* **2024**, *53*, 102812.
20. Santalla-Hernández, Á.; Naveiro-Fuentes, M.; López-Criado, M.S.; Naveiro-Flores, R.; Fernández-Parra, J. Clinical outcomes after 2-year follow-up of transvaginal radiofrequency ablation of symptomatic uterine fibroids. *J. Obstet. Gynaecol. Res.* **2025**, *51*, e16216.
21. Rey, V.E.; Falcon, M.M.; Ferrara, I.; Yanes, G. Pregnancy outcomes after transvaginal radiofrequency ablation of leiomyomas. *Obstet. Gynecol.* **2025**, *145*, 346–353.
22. Liu, L.; Wang, T.; Lei, B. High-intensity focused ultrasound (HIFU) ablation versus surgical interventions for symptomatic uterine fibroids: A meta-analysis. *Eur. Radiol.* **2022**, *32*, 1195–1204. https://doi.org/10.1007/s00330-021-08156-6.

23. Krzyzanowski, J.; Wozniak, S.; Szkodziak, P.; Krzyzanowski, A.; Wojciech, W.; Paszkowski, T. Minimally invasive treatment options for uterine fibroids—State of the art 2021. *Ginekol. Pol.* **2022**, *93*, 242–247. https://doi.org/10.5603/GP.a2021.0202.
24. Wu, L.; Zhong, L.; Zheng, Q.; Huang, L.; Lin, F.; Chen, Z.; Lian, C. Application of high intensity focused ultrasound in the treatment of uterine fibroids in overweight/obese women. *BMC Womens Health* **2025**, *25*, 243. https://doi.org/10.1186/s12905-025-03797-x.
25. Tsai, M.C.; Chang, L.T.; Tam, K.W. Comparison of high-intensity focused ultrasound and conventional surgery for patients with uterine myomas: A systematic review and meta-analysis. *J. Minim. Invasive Gynecol.* **2021**, *28*, 1712–1724. https://doi.org/10.1016/j.jmig.2021.06.002.
26. Madueke-Laveaux, O.S.; Elsharoud, A.; Al-Hendy, A. Long-term risks of hysterectomy for benign indication—A systematic review. *J. Clin. Med.* **2021**, *10*, 5335. https://doi.org/10.3390/jcm10225335.
27. Shahzadi, M.; Jamil, U.; Hanif, S.; Ullah, I.; Shahbaz, S.; Akhtar, A.; Ullah, T.R.; Dar, S.H. Intra-operative and post-operative common complications of hysterectomy. *J. Health Wellness Community Res.* **2026**, *4*, e1235.
28. Deipolyi, A.R.; Annie, F.; Bush, S.H., 2nd; Spies, J. Hysterectomy and myomectomy versus uterine artery embolization for symptomatic fibroids and adenomyosis: Trends and adverse events in 70,000 patients. *J. Vasc. Interv. Radiol.* **2025**, *36*, 1011–1018. https://doi.org/10.1016/j.jvir.2025.02.026.
29. Lenfant, L.; Canlorbe, G.; Belghiti, J.; Kreaden, U.S.; Hebert, A.E.; Nikpayam, M.; Uzan, C.; Azaïs, H. Robotic-assisted benign hysterectomy compared with laparoscopic, vaginal, and open surgery: A systematic review and meta-analysis. *J. Robot. Surg.* **2023**, *17*, 2647–2662. https://doi.org/10.1007/s11701-023-01724-6.
30. Guarnaccia, M.M.; Rein, M.S. Traditional surgical approaches to uterine fibroids: Abdominal myomectomy and hysterectomy. *Clin. Obstet. Gynecol.* **2001**, *44*, 385–400.
31. Ruiz, P.; Papi, J.S.C.; de Jesus, A.K.P.; Cagnani Alves, P.A.L. Comparison between abdominal, vaginal, and laparoscopic hysterectomy: Advances, indications and outcomes. *Asclepius Int. J. Sci. Health Sci.* **2025**, *4*, 154–161.
32. Antoun, L.; Woolley, R.; Middleton, L.; Smith, P.; Saridogan, E.; Cooper, K.; McKinnon, W.; Bevan, S.; Ziomek, K.; Sairally, Z.; et al. Comparison of complications and recovery after laparoscopic and abdominal hysterectomy (LAVA trial). *BMJ Open* **2025**, *15*, e096265. https://doi.org/10.1136/bmjopen-2024-096265.
33. Lethaby, A.; Vollenhoven, B.; Sowter, M. Efficacy of pre-operative gonadotrophin hormone releasing analogues for women with uterine fibroids undergoing hysterectomy or myomectomy: A systematic review. *BJOG: Int. J. Obstet. Gynaecol.* **2002**, *109*, 1097–1108.
34. Lethaby, A.; Vollenhoven, B.; Sowter, M. Pre-operative GnRH analogue therapy before hysterectomy or myomectomy for uterine fibroids. *Cochrane Database Syst. Rev.* **2001**, *2*, CD000547.
35. Azadi, A.M.; Masoud, A.T.; Ulibarri, H.B.; Arroyo, A.B.; Coriell, C.B.; Goetz, S.B.; Moir, C.B.; Moberly, A.B.; Gonzalez, D.B.; Blanco, M.B.; et al. Vaginal hysterectomy compared with laparoscopic hysterectomy in benign gynecologic conditions: A systematic review and meta-analysis. *Obstet. Gynecol.* **2023**, *142*, 1373–1394. https://doi.org/10.1097/AOG.0000000000005434.
36. Landeen, L.B.; Bell, M.C.; Hubert, H.B.; Bennis, L.Y.; Knutsen-Larson, S.S.; Seshadri-Kreaden, U. Clinical and cost comparisons for hysterectomy via abdominal, laparoscopic, vaginal and robot-assisted approaches. *South Dak. Med.* **2011**, *64*, 197.
37. Tapper, A.M.; Hannola, M.; Zeitlin, R.; Isojärvi, J.; Sintonen, H.; Ikonen, T.S. Cost analysis of robot-assisted hysterectomy in malignant and benign conditions. *Eur. J. Obstet. Gynecol. Reprod. Biol.* **2014**, *177*, 1–10.
38. Horng, H.C.; Wen, K.C.; Su, W.H.; Chen, C.S.; Wang, P.H. Review of myomectomy. *Taiwan J. Obstet. Gynecol.* **2012**, *51*, 7–11.
39. Cianci, S.; Gulino, F.A.; Palmara, V.; La Verde, M.; Ronsini, C.; Romeo, P.; Occhipinti, S.; Incognito, G.G.; Capozzi, V.A.; Restaino, S.; et al. Exploring surgical strategies for uterine fibroid treatment: A comprehensive review of literature on open and minimally invasive approaches. *Medicina* **2024**, *60*, 64. https://doi.org/10.3390/medicina60010064.
40. Goldberg, J.; Pereira, L. Pregnancy outcomes following treatment for fibroids: Uterine fibroid embolization versus laparoscopic myomectomy. *Curr. Opin. Obstet. Gynecol.* **2006**, *18*, 402–406.

41. Paredes, J.S.; Lee, C.L.; Chua, P.T. Myomectomy: Choosing the surgical approach—A systematic review. *Gynecol. Minim. Invasive Ther.* **2024**, *13*, 146–153. https://doi.org/10.4103/gmit.gmit_152_23.
42. Majak, G.B.; Lieng, M.; Qvigstad, E. Clinical outcome after laparoscopic and open abdominal myomectomy. *Gynecol. Surg.* **2012**, *9*, 231–234.
43. Gambacorti-Passerini, Z.; Gimovsky, A.C.; Locatelli, A.; Berghella, V. Trial of labor after myomectomy and uterine rupture: A systematic review. *Acta Obstet. Gynecol. Scand.* **2016**, *95*, 724–734.
44. Claeys, J.; Hellendoorn, I.; Hamerlynck, T.; Bosteels, J.; Weyers, S. Risk of uterine rupture after myomectomy: A systematic review and meta-analysis. *Gynecol. Surg.* **2014**, *11*, 197–206.
45. Sznurkowski, J.J.; Wnuk, J. Feasibility of laparoscopic removal of the largest documented uterine fibroid without morcellation. *Reports* **2025**, *8*, 71.
46. Chene, G.; Miquel, L.; Agostini, A.; Bendifallah, S.; Solignac, C.; Darne, B.; Languille, S.; Timoh, K.N.; Carbonnel, M. Safety of in-bag morcellation during laparoscopic myomectomy and hysterectomy: Systematic review and meta-analysis. *J. Minim. Invasive Gynecol.* **2026**, *33*, 164–177.
47. Mourad, A.; Kamga-Ngande, C.; Albaini, O.; Antaki, R. Robotic surgery in myomectomies: Systematic review and meta-analysis. *J. Robot. Surg.* **2024**, *18*, 184.
48. Sheng, Y.; Hong, Z.; Wang, J.; Mao, B.; Wu, Z.; Gou, Y.; Zhao, J. Robot-assisted versus laparoscopic myomectomy: Systematic evaluation and meta-analysis. *World J. Surg. Oncol.* **2023**, *21*, 230.
49. Alghamdi, H.A.; Al-Zahrani, M.; Alkodae, S.; Alzeer, R.; Alharbi, R.; Althafar, J.; Asulaimani, M.; Elbashir, A.E. Comparative analysis of robotic-assisted laparoscopic myomectomy versus traditional abdominal and laparoscopic myomectomy: A meta analysis and systematic review. *Afr. J. Reprod. Health* **2025**, *29*, 230–246.
50. Di Spiezio Sardo, A.; Mazzon, I.; Bramante, S.; Bettocchi, S.; Bifulco, G.; Guida, M.; Nappi, C. Hysteroscopic myomectomy: Review of surgical techniques. *Hum. Reprod. Update* **2008**, *14*, 101–119.
51. Lasmar, R.B.; Lasmar, B.P.; Moawad, N.S. Hysteroscopic myomectomy. *Medicina* **2022**, *58*, 1627.
52. Vilos, G.A.; Allaire, C.; Laberge, P.Y.; Leyland, N.; Vilos, A.G.; Murji, A.; Chen, I. The management of uterine leiomyomas. *J. Obstet. Gynaecol. Can.* **2015**, *37*, 157–178. https://doi.org/10.1016/S1701-2163(15)30338-8.
53. Maruo, T.; Ohara, N.; Yoshida, S.; Nakabayashi, K.; Sasaki, H.; Xu, Q.; Chen, W.; Yamada, H. Translational research with progesterone receptor modulator motivated by the use of levonorgestrel-releasing intrauterine system. *Contraception* **2010**, *82*, 435–441.
54. Sangkomkamhang, U.S.; Lumbiganon, P.; Laopaiboon, M.; Mol, B.W. Progestogens or progestogen-releasing intrauterine systems for uterine fibroids. *Cochrane Database Syst. Rev.* **2020**, *2020*, CD008994. https://doi.org/10.1002/14651858.CD008994.pub3.
55. Donnez, J. Uterine Fibroids and Progestogen Treatment: Lack of Evidence of Its Efficacy: A Review. *J. Clin. Med.* **2020**, *9*, 3948. https://doi.org/10.3390/jcm9123948.
56. Ciebiera, M.; Madueke-Laveaux, O.S.; Feduniw, S.; Ulin, M.; Spaczyński, R.; Zgliczyńska, M.; Bączkowska, M.; Zarychta, E.; Łoziński, T.; Ali, M.; et al. GnRH agonists and antagonists in therapy of symptomatic uterine fibroids—Current roles and future perspectives. *Expert Opin. Pharmacother.* **2023**, *24*, 1799–1809. https://doi.org/10.1080/14656566.2023.2248890.
57. Martín, M.J.S.; López, C.H.; García, I.C.; Quevedo, I.C. Efficacy of GnRH antagonists in the treatment of uterine fibroids: A meta-analysis. *Arch. Gynecol. Obstet.* **2025**, *311*, 685–696. https://doi.org/10.1007/s00404-025-07932-9.
58. Murji, A.; Whitaker, L.; Chow, T.L.; Sobel, M.L. Selective progesterone receptor modulators (SPRMs) for uterine fibroids. *Cochrane Database Syst. Rev.* **2017**, *4*, CD010770. https://doi.org/10.1002/14651858.CD010770.pub2.
59. Woźniak, S.; Pietrzak, B.; Paszkowski, T.; Radowicki, S.; Pawelczyk, L.; Wielgoś, M. Farmakoterapia mięśniaków macicy. *Ginekol. Perinatol. Prakt.* **2017**, *2*, 43–47, doi:10.5603/gipp.50747.
60. Deng, L.; Wu, T.; Chen, X.Y.; Xie, L.; Yang, J. Selective estrogen receptor modulators (SERMs) for uterine leiomyomas. *Cochrane Database Syst. Rev.* **2012**, *10*, CD005287. https://doi.org/10.1002/14651858.CD005287.pub4.

61. Song, H.; Lu, D.; Navaratnam, K.; Shi, G. Aromatase inhibitors for uterine fibroids. *Cochrane Database Syst. Rev.* **2013**, *10*, CD009505. https://doi.org/10.1002/14651858.CD009505.pub2.
62. Blair, H.A. Relugolix/Estradiol/Norethisterone Acetate: A Review in Endometriosis-Associated Pain. *Drugs* **2024**, *84*, 449–457. https://doi.org/10.1007/s40265-024-02018-3.
63. European Medicines Agency. Ryeqo 40 mg/1 mg/0.5 mg Film-Coated Tablets: EU Summary of Product Characteristics. Available online: https://www.ema.europa.eu/en/documents/product-information/ryeqo-epar-product-information_en.pdf (accessed on 21 September 2022).
64. Chmaj-Wierzchowska, K.; Lach, A.; Bednarek, K.; Nowak, A.; Mruczyński, A.; Bruszewski, A.; Piekarski, P.; Malinger, A.; Wilczak, M. Transvaginal uterine fibroid radiofrequency ablation (TV-RFA): Retrospective analysis and preliminary report. *Life* **2025**, *15*, 1841. https://doi.org/10.3390/life15121841.
65. Piriyev, E.; Dieter, A.; Schiermeier, S.; Renner, S.P.; Römer, T. Combined transcervical radiofrequency ablation and hysteroscopic myomectomy: Expanding treatment to diverse fibroid types. *Arch. Gynecol. Obstet.* **2025**, *312*, 1247–1252. https://doi.org/10.1007/s00404-025-08127-y.
66. Shifrin, G.; Engelhardt, M.; Gee, P.; Pschadka, G. Transcervical fibroid ablation with the Sonata system for treatment of submucous and large uterine fibroids. *Int. J. Gynaecol. Obstet.* **2021**, *155*, 79–85. https://doi.org/10.1002/ijgo.13638.
67. Miller, C.E.; Osman, K.M. Transcervical radiofrequency ablation of symptomatic uterine fibroids: 2-year results of the SONATA pivotal trial. *J. Gynecol. Surg.* **2019**, *35*, 345–349. https://doi.org/10.1089/gyn.2019.0012.
68. Hansen-Lindner, L.; Schmid-Lossberg, J.; Toub, D. Transcervical fibroid ablation (TFA): Pregnancy outcomes. *J. Minim. Invasive Gynecol.* **2024**, *31*, S13. https://doi.org/10.1016/j.jmig.2024.09.045.
69. Christoffel, L.; Bends, R.; Toub, D.; Schiermeier, S.; Pschadka, G.; Engelhardt, M.; Quinn, S.; Hartmann, M.; Habiba, M.; Felberbaum, R.; et al. Pregnancy outcomes after transcervical radiofrequency ablation of uterine fibroids with the Sonata system. *J. Gynecol. Surg.* **2022**, *38*, 207–213. https://doi.org/10.1089/gyn.2021.0136.
70. Wray, J.K.; Dixon, B.; Przkora, R. Radiofrequency ablation. In *StatPearls [Internet]*; StatPearls Publishing: Treasure Island, FL, USA, 2025.
71. Firouznia, K.; Ghanaati, H.; Jalali, A.H.; Shakiba, M. Uterine artery embolization for symptomatic fibroids: A review of evidence. *Iran. Red Crescent Med. J.* **2013**, *15*, e16699. https://doi.org/10.5812/ircmj.16699.
72. Szkodziak, P.; Pyra, K.; Szkodziak, F.; Krzyżanowski, J.; Czuczwar, P.; Woźniak, S.; Jargiełło, T.; Paszkowski, T. The Lublin protocol of uterine artery embolization in the treatment of symptomatic uterine fibroids. *J. Vis. Exp.* **2020**, *163*, e61530. https://doi.org/10.3791/61530.
73. Kröncke, T.; David, M. Uterine artery embolization (UAE) for fibroid treatment: Results of the 7th radiological gynecological expert meeting. *Geburtshilfe Frauenheilkd.* **2019**, *79*, 688–692. https://doi.org/10.1055/a-0893-4807.
74. Marshburn, P.B.; Matthews, M.L.; Hurst, B.S. Uterine artery embolization as a treatment option for uterine myomas. *Obstet. Gynecol. Clin. North Am.* **2006**, *33*, 125–144. https://doi.org/10.1016/j.ogc.2005.12.009.
75. Bellala, P.; Valakkada, J.; Ayyappan, A.; Kumar, S. Evidences in uterine artery embolization: A radiologist's primer. *J. Clin. Interv. Radiol. ISVIR* **2023**, *7*, 87–96. https://doi.org/10.1055/s-0042-1758050.
76. Waldron, M.G.; Kassamani, Y.W.; O'mahony, A.T.; O'mahony, S.M.; O'sullivan, O.E.; Power, S.P.; Spence, L.; Maher, M.M.; O'connor, O.J.; Buckley, M.M. Uterine artery embolisation and post-embolisation syndrome: A systematic review. *Diagnostics* **2022**, *12*, 2916. https://doi.org/10.3390/diagnostics12122916.
77. Czuczwar, P.; Stępniak, A.; Wrona, W.; Woźniak, S.; Milart, P.; Paszkowski, T. Influence of uterine artery embolisation on ovarian reserve, fertility, and pregnancy outcomes. *Prz. Menopauzalny* **2016**, *15*, 205–209. https://doi.org/10.5114/pm.2016.65665.
78. Loddo, A.; Djokovic, D.; Drizi, A.; De Vree, B.P.; Sedrati, A.; van Herendael, B.J. Hysteroscopic myomectomy: The guidelines of the International Society for Gynecologic Endoscopy (ISGE). *Eur. J. Obstet. Gynecol. Reprod. Biol.* **2022**, *268*, 121–128. https://doi.org/10.1016/j.ejogrb.2021.11.434.
79. Munro, M.G.; Critchley, H.O.; Fraser, I.S. The FIGO classification of causes of abnormal uterine bleeding in the reproductive years. *Fertil. Steril.* **2011**, *95*, 2204–2208. https://doi.org/10.1016/j.fertnstert.2011.03.079.
80. Piecak, K.; Milart, P. Hysteroscopic myomectomy. *Prz. Menopauzalny* **2017**, *16*, 126–128. https://doi.org/10.5114/pm.2017.72757.

81. Lima, M.P.J.S.; Costa-Paiva, L.; Brito, L.G.O.; Baccaro, L.F. Factors associated with complications of hysteroscopic myomectomy. *Rev. Bras. Ginecol. Obstet.* **2020**, *42*, 476–485. https://doi.org/10.1055/s-0040-1713915.
82. Zhang, W.; French, H.; O'Brien, M.; Movilla, P.; Isaacson, K.; Morris, S. Incidence of intrauterine adhesions after hysteroscopic myomectomy in patients seeking fertility. *J. Minim. Invasive Gynecol.* **2023**, *30*, 805–812. https://doi.org/10.1016/j.jmig.2023.05.013.
83. Donnez, J.; Tatarchuk, T.F.; Bouchard, P.; Puscasiu, L.; Zakharenko, N.F.; Ivanova, T.; Ugocsai, G.; Mara, M.; Jilla, M.P.; Bestel, E.; et al. Ulipristal acetate versus placebo for fibroid treatment before surgery. *New Engl. J. Med.* **2012**, *366*, 409–420. https://doi.org/10.1056/NEJMoa1103182.
84. American College of Obstetricians and Gynecologists. Management of symptomatic uterine leiomyomas: ACOG practice bulletin No. 228. *Obstet. Gynecol.* **2021**, *137*, e100–e115. https://doi.org/10.1097/AOG.0000000000004401.
85. Altgassen, C.; Kuss, S.; Berger, U.; Löning, M.; Diedrich, K.; Schneider, A. Complications in laparoscopic myomectomy. *Surg. Endosc.* **2006**, *20*, 614–618. https://doi.org/10.1007/s00464-004-2181-8.
86. Perelli, F.; Tinelli, A. Minimally invasive myomectomy. In *The Global Library of Women's Medicine (GLOWM)*; Arulkumaran, S., Regan, L., Eds.; ISGE: London, UK, 2019.
87. National Institute for Health and Care Excellence. Magnetic Resonance Image-Guided Transcutaneous Focused Ultrasound for Uterine Fibroids (IPG413); NICE: London, UK, 2011.
88. Roberts, A. Magnetic resonance-guided focused ultrasound for uterine fibroids. *Semin. Intervent. Radiol.* **2008**, *25*, 394–405. https://doi.org/10.1055/s-0028-1102999.
89. Yu, L.; Zhu, S.; Zhang, H.; Wang, A.; Sun, G.; Liang, J.; Wang, X. Efficacy and safety of MR-HIFU and US-HIFU in treating uterine fibroids <300 cm^3: A meta-analysis. *Int. J. Hyperth.* **2021**, *38*, 1126–1132. https://doi.org/10.1080/02656736.2021.1954245.
90. Koga, K.; Fukui, M.; Fujisawa, M.; Suzukamo, Y. Impact of diagnosis and treatment of uterine fibroids on quality of life and labor productivity: The Japanese online survey for uterine fibroids and quality of life (JOYFUL survey). *J. Obstet. Gynaecol. Res.* **2023**, *49*, 2528–2537.
91. Hervé, F.; Katty, A.; Isabelle, Q.; Céline, S. Impact of uterine fibroids on quality of life: A national cross-sectional survey. *Eur. J. Obstet. Gynecol. Reprod. Biol.* **2018**, *229*, 32–37.
92. de Smit, N.S.; de Lange, M.E.; Boomsma, M.F.; Huirne, J.A.F.; Hehenkamp, W.J.K. Current treatment for symptomatic uterine fibroids: Available evidence and therapeutic dilemmas. *Lancet* **2025**, *406*, 91–102.
93. Anchan, R.M.; Spies, J.B.; Zhang, S.; Wojdyla, D.; Bortoletto, P.; Terry, K.; Disler, E.; Milne, A.; Gargiulo, A.; Petrozza, J.; et al. Long-term health-related quality of life and symptom severity following hysterectomy, myomectomy, or uterine artery embolization for the treatment of symptomatic uterine fibroids. *Am. J. Obstet. Gynecol.* **2023**, *229*, 275.e1–275.e17.
94. Borah, B.J.; Yao, X.; Laughlin-Tommaso, S.K.; Heien, H.C.; Stewart, E.A. Comparative effectiveness of uterine leiomyoma procedures using a large insurance claims database. *Obstet. Gynecol.* **2017**, *130*, 1047–1056.
95. Babalola, O.; Gebben, D.; Tarver, M.E.; Sangha, R.; Roberts, J.; Price, V. Patient preferences regarding surgical treatment methods for symptomatic uterine fibroids. *Ther. Innov. Regul. Sci.* **2023**, *57*, 976–986.
96. Riggan, K.A.; Stewart, E.A.; Balls-Berry, J.E.; Venable, S.; Allyse, M.A. Patient recommendations for shared decision-making in uterine fibroid treatment decisions. *J. Patient Exp.* **2021**, *8*, 23743735211049655.
97. None, D.N.W.; None, D.M.S.; None, D.B.Z.B.; None, D.S.R. Medical versus Surgical Management of Symptomatic Uterine Fibroids: A Systematic Review of Clinical Outcomes, Fertility, Safety, and Reintervention. *Int. J. Med. Pharm. Res.* **2026**, *7*, 775-782.
